# Osseodensification over standard drilling methods on implant stability and crestal bone level

**DOI:** 10.6026/973206300214605

**Published:** 2025-12-15

**Authors:** Abhishek Mehta, Saloni Vijaywargiya, Pankaj Jindal, Sandeep Kalra, Gopal Krishna Choudhury, Jeevan Shetty, Saptadisha Rakshit, Sambit Prasad, Nitin Purohit, Savita Singh

**Affiliations:** 1 Department of Conservative Dentistry and Endodontic, Daswani Dental College and Research Centre, Kota, Rajasthan, India; 2 Department of Oral and Maxillofacial Surgery, Pacific Dental College and Research Centre, Udaipur, Rajasthan, India; 3 Department of Prosthodontics, Maharishi Markandeshwar College of Dental Sciences and Research, (MMCDSR), Mullana, Haryana, India; 4 Department of Prosthodontics, Institute of Dental Sciences, S "O" A Deemed to be University, Bhubaneswar, Odisha, India; 5 Department of Periodontics and Implantology, KGF College of Dental Sciences, KGF, Karnataka, India; 6 Intern, Kalinga Institute of Dental Sciences, KIIT Deemed to be University, Patia, Bhubaneswar-751024, Odisha, India; 7 Dental Surgeon, Community Health Centre, Odagaon, Nayagarh, Odisha, India; 8 Department of Dentistry, District Hospital, Rudraprayag, Uttarakhand, India; 9 Private Practitioner, Department of Oral and maxillofacial Pathology and Microbiology, New Delhi, India

**Keywords:** Insertion torque, osseodensification, standard drilling

## Abstract

Primary implant stability basically depends on the quality and the quantity of bone. Osseodensification is a non-excavating additive drilling sequence used to enhance implant stability. Therefore, it is of interest to compare insertion torque (IT) and implant stability quotient (ISQ) values after 6 months after surgery for standard drilling (SD) and osseodensification (OD) drilling methods. In this prospective study, total 40 implants with the same diameter and length were placed in 30 patients using SD (20 implants) and OD (20 implants) methods in the posterior maxilla. The amounts of IT after surgery, ISQ and crestal bone level at baseline and six months later were evaluated. The obtained data was statically evaluated. The OD technique provided a higher IT rate after surgery compared to the SD method.

## Background:

Nowadays, the best way to replace lost teeth is with dental implants [1, 2, 3, 4, 5, 6-7]. The alveolar ridge is a crucial anatomical feature for dental implants to be successful [8]. Primary and secondary implant stability is critical for the outcome of implant rehabilitation. Several methods, including undersize osteotomy preparation, osteotome-based bone preparation (Summer's Technique), and osseodensification, can improve primary implant stability [9]. During the preparation of the osteotomy site, autogenous bone is lost due to the subtractive nature of the conventional drilling routine. A non-excavating additive drilling technique called osseodensification is utilized to improve implant stability [10]. The success of the implant's primary stability is also correlated with the patient's bone density, resonance frequency analysis (RFA), and insertion torque (IT). It is stated that one viable technique for assessing implant stability is resonance frequency analysis (RFA) [11, 12]. Over time as a result of the implant's surrounding bone changing, clinically, implant stability quotients (ISQ) can be obtained using resonant frequency analysis or insertion torque (IT) measurements utilizing surgical handpieces to evaluate the degree of implant stability [13]. For predicted osseointegration and earlier loading, IT values > 35 Ncm or ISQ values >68 have been deemed appropriate [14]. Following implant placement, these values can be attained and sustained throughout the initial phase of osseointegration [15]. The primary stability of dental implants is mostly determined by factors such bone density, surgical technique, and implant configuration [16]. Increased initial bone-to-implant contact from higher insertion torque improves primary stability [8]. Osseodensification (OD), a novel approach to osteotomy preparation, makes use of specially made burs that permit bone condensation and preservation through compaction autografting during osteotomy preparation, hence improving primary implant stability [17]. In this method, counterclockwise drills, which are designed to improve bone density, are among the newest equipment available. They are also as efficient as traditional drills in a clockwise orientation. A non-subtractive technique used in OD drills expands the implant site and boosts bone density [10]. This OD method preserves bone and maintains density by utilizing the elastic and plastic characteristics of bone. An osseous material is autografted into the trabecular space as a result of bone mass preservation and compaction [15].

When a foreign body challenges bone metabolism by causing stress or strain in the peri-implant tissue and triggering highly integrated and complex immunomodulated inflammatory reactions, osseointegration is the formation of a structural and functional bone-to-implant interface without soft tissue interference, ultimately resulting in the formation of new bone [18]. Normally undersized osteotomies are used to optimize the first bone-to-implant contact in order to promote primary stability. However, ischemia and severe bone compression could result from this. Piezosurgical osteotomies were created as a solution to this issue. In order to preserve bone density and prepare the implant site with the least amount of stress, osseodensification uses autologous lateral bone compaction to generate an implant osteotomy with improved bone density. Over time, this would increase insertion torque and stability [8]. The type of flap procedure during implant placement can affects crestal bone level [19]. There is no data for changes in crestal bone levels using the conventional over-osseodensification approach of implant placement. Therefore, it is of interest to evaluate crestal bone alterations and compare insertion torque (IT) and implant stability quotient (ISQ) values with standard drilling (SD) and osseodensification (OD) drilling methods both immediately and six months after surgery.

## Materials and Methods:

This study was done in the department of Prosthodontics after the approval from the institutional ethics committee and informed consent from the participants. This prospective observation study was done to investigate the influence of two osteotomy techniques. A two-sample t-test (PASS2) was used to determine the minimal sample size in order to obtain a 5% α error and 20% β error within an effect size of 18 at n = 8 implants. This was then increased by 20% to account for anticipated losses and refusals. For each factor (20 OD, 20 SD), about 20 implants were needed per group. The study was conducted between February 2023 and August 2024 on patients who required dental implants in the posterior maxilla. After taking into account the inclusion and exclusion criteria, all patients were enlisted, and demographic information was gathered. Inclusion criteria were; healthy individuals between the ages of 20 and 40 who had enough residual bone volume for implant insertion without the requirement for bone augmentation, a minimum ridge height and width of 9 and 6 mm and at least six months following extraction. Cone-beam CT scans were performed on each patient. In this prospective study, 30 patients in two groups-Group I OD Osseodensification/test group (20 implants) and Group II conventional drilling/control method (20 implants)-had total 40 implants placed in their posterior maxilla with the same diameter and length. The final implant was placed after the osteotomy to the required depth (3.75 mm diameter) for Group I (conventional drilling technique). For Group II, the pilot drill was utilized to prepare the osteotomy to the required depth, later osseodensification drills were employed sequentially in accordance with the implant diameter protocol, and the drilling was done in a clockwise manner before the final implant placement. Full-thickness surgical flaps were raised following local anathesis and under-aspect surgery, and implant osteotomies were carried out using a maximum torque of 50 Ncm either by SD or in accordance with particular implant company procedures. Implants used were 3.75 x 10 in diameter and 1 mm subcrestal in both groups. When the maximal torque value (Ncm) approached the implant insertion termination, IT data were noted. Following six months of recovery, ISQ values were also noted during follow-up appointments. Using CBCT, the crestal bone levels surrounding the implant were assessed at baseline and six months later. Using SPSS statistical software version 24.0, the acquired data was statistically assessed using an ANOVA test with P<0.05.

## Results:

Following surgery, the OD drilling method produced 35% more IT than the SD drilling method (P < 0.001). Following surgery, there was no significant difference between SD and OD in ISQ values (P1 < 0.165). The ISQ results did not differ significantly between the two groups six months following surgery. ISQ scores for every experimental parameter in the OD and SD groups stayed over the 66 threshold. At baseline, IT values and ISQ values were positively correlated (Table 1 - see PDF). In every instance, the crestal bone levels demonstrated good bone growth, with Group I's levels being marginally greater than Group II's (Table 2 - see PDF).

## Discussion:

For osseointegration to be successful, surgical instruments that increase implant stability in the alveolar bone must be chosen [20]. To get around the shortcomings of the conventional procedure, a different strategy has been designed. It has been suggested that an OD drilling process maintains bone by compacting the particles into the osteotomy wall, as opposed to the removal of bone particles in traditional SD procedures [10]. By comparing consecutive measurements at predetermined intervals, ISQ is an effective indicator [15]. OD was found to increase primary and secondary stability as well as insertion and removal torque as compared to traditional drilling [21]. This study evaluated the impact of SD and OD techniques on IT and ISQ both immediately following surgery and six months following implant implantation. This study shown that, by forming a layer of mineralized bone around the osteotomy site, the OD method boosts the primary stability and bone volume on the implant surface. When an osteotomy is successful, the implant is positioned in three dimensions with the proper biomechanical stability. The direct contact between the implant's surface and the bone, which minimizes micromotion at this interface, is essential to the stability of the implant [15]. Two methods are used by OD to maintain the bone: autograft densification of bone particles throughout the length and apex of the osteotomy and densification of cancellous bone via viscoelastic and plastic deformation [10]. Because of the spring-back action, OD offers a balance between preserving bone volume and increased implant stability [15]. In a study comparing OD and SD, Bergamo and colleagues found that OD improved the implant's main stability and bone density. In contrast to the current study, ISQ considerably rose in OD as opposed to SD [22]. Huwais and Meyer looked into how OD affected bone density, bone-to-implant contact, and primary stability. There were no discernible variations in ISQ across the three groups [10]. Mello-Machado *et al.* used OD and SD methods to compare implant stability in low-quality bone. After six months, ISQ levels were higher and implant survival was comparable in both groups [23]. According to Moghaddas *et al.*'s research, the OD technique had a greater IT rate following surgery than the SD method, although there was no discernible difference between the two groups [15]. According to Shanmugam *et al.* the osseodensification process increased the width at the apex as well as the radiographic bone density [8] Aloorker *et al.* reported increased radiographic bone density surrounding the implant following OD in the maxillary posterior region, supporting the benefit of OD [24]. According to Pereira *et al.* when compared to traditional subtractive osteotomy, OD has no detrimental effects on osseointegration [19]. Implant stability may benefit by the OD method, especially in areas with low-to-moderate bone density [25]. Hindi *et al.* used CBCT to assess the impact of implant site preparation in low-density bone utilizing the osseodensification method in terms of implant stability changes during the osseous healing period and peri-implant bone density. They come to the conclusion that while osseodensification enhanced peri-implant bone density and produced high primary stability, it was unable to stop the decline in implant stability over the first six weeks following implant implantation [21]. Compared to trabecular bone, it has been discovered that cortical bone-particularly high-density cortical bone-significantly contributes to primary implant stability [26]. Sultana *et al.* discovered no statistically significant difference between OD drilling and conventional drilling in terms of crestal bone levels and implant durability [11]. The largest bone quality were detected in the anterior mandible, anterior maxilla, posterior mandible, and posterior maxilla, according to Singh *et al.* [27] Manas *et al.* used the Osstell® and AnyCheck® procedures to test the stability of primary and secondary implants, and they found that both approaches performed similarly [28]. According to Althobaiti *et al.* osseodensification drilling outperformed traditional drilling techniques in terms of resonance frequency analysis (RFA) and ISQ values [29]. Osseodensification is a method that shows considerable potential in the area of dental implants. Compared to conventional drilling, it demonstrates enhanced torque values, bone development, and stability [30, 31]. Bone quality and type of insertion torque helps in predicting the success of dental implant. We discovered that increased peri-implant bone density and good primary stability were the outcomes of osseodensification. To confirm the findings, more research is required.

## Conclusion:

OD was successful in improving and increasing the stability of the implant by maintaining bone structure and volume and increasing bone density compared to the SD method. This allowed the implant to be passively placed in the prepared site without causing stress despite its high stability.
